# The Role of Water Channel Proteins in Facilitating Recovery of Leaf Hydraulic Conductance from Water Stress in *Populus trichocarpa*

**DOI:** 10.1371/journal.pone.0111751

**Published:** 2014-11-18

**Authors:** Joan Laur, Uwe G. Hacke

**Affiliations:** Department of Renewable Resources, University of Alberta, 442 Earth Sciences Building, Edmonton, Alberta, Canada; Università della Calabria, Italy

## Abstract

Gas exchange is constrained by the whole-plant hydraulic conductance (*K*_plant_). Leaves account for an important fraction of *K*_plant_ and may therefore represent a major determinant of plant productivity. Leaf hydraulic conductance (*K*_leaf_) decreases with increasing water stress, which is due to xylem embolism in leaf veins and/or the properties of the extra-xylary pathway. Water flow through living tissues is facilitated and regulated by water channel proteins called aquaporins (AQPs). Here we assessed changes in the hydraulic conductance of *Populus trichocarpa* leaves during a dehydration-rewatering episode. While leaves were highly sensitive to drought, *K*_leaf_ recovered only 2 hours after plants were rewatered. Recovery of *K*_leaf_ was absent when excised leaves were bench-dried and subsequently xylem-perfused with a solution containing AQP inhibitors. We examined the expression patterns of 12 highly expressed AQP genes during a dehydration-rehydration episode to identify isoforms that may be involved in leaf hydraulic adjustments. Among the AQPs tested, several genes encoding tonoplast intrinsic proteins (TIPs) showed large increases in expression in rehydrated leaves, suggesting that TIPs contribute to reversing drought-induced reductions in *K*_leaf_. TIPs were localized in xylem parenchyma, consistent with a role in facilitating water exchange between xylem vessels and adjacent living cells. Dye uptake experiments suggested that reversible embolism formation in minor leaf veins contributed to the observed changes in *K*_leaf_.

## Introduction

High gas exchange rates can only be sustained when leaves are kept well hydrated. This, in turn, depends on the properties of the xylem pipeline and on the way in which water moves through living cells in roots and leaves [1], [2]. Leaf hydraulic conductance is emerging as an important component of whole-plant hydraulic conductance [3]–[7]. Like in roots and stems, the hydraulic conductance of leaves declines as the water potential becomes more negative. This loss of hydraulic conductance is due to embolism formation in leaf veins [8], [9], cell shrinkage [10], collapse of xylem conduits [11], and/or to decline in the permeability of extra-xylary tissues [12]. Compared with stems, leaves [13] and roots [14] are often more vulnerable to hydraulic dysfunction. In some cases, however, the hydraulic conductance of these plant organs may also be able to quickly recover from the effects of drought [3], [15].

This recovery of hydraulic function may be facilitated by the activity of aquaporin (AQP) water channels [16]–[20]. AQPs belong to the major intrinsic protein (MIP) superfamily, a family of protein pores present in the membranes of almost all biological cells to facilitate the diffusion of a wide range of small uncharged solutes. Plant MIPs form a particularly large family of proteins, with 28 members in *Vitis vinifera* [21], ≥30 members in *Arabidopsis thaliana*, *Picea glauca* and *Oryza sativa* [19], [22], [23], and >50 members in *Populus trichocarpa* [24]. The plant-specific plasma membrane intrinsic proteins (PIPs), with their highly conserved phylogenetic subgroups PIP1 and PIP2, and tonoplast intrinsic proteins (TIPs) show significant water transport activity *in* *vitro* and *in planta* [25]–[27]. Regulation of AQPs via transcription, translation, post-translational modifications or trafficking allows plant cells and organs to respond to hydraulic changes in their surrounding environment [28].

In this present study, *Populus trichocarpa* plants were exposed to moderate drought and then rewatered. The objective was to study the recovery of *K*_leaf_ from water stress at both physiological and molecular levels. We hypothesized that leaves would quickly (i.e., within hours) recover from water stress, and that this would be associated with modulation of AQP activity. To test this hypothesis, we monitored *K*_leaf_ and Ψ_leaf_ during a dehydration-rehydration episode. We also explored the regulation of 12 leaf-expressed *AQP* isoforms as well as the tissue-specific location of PIP1, PIP2 and TIP2 proteins. Recovery of *K*_leaf_ was assessed in two ways: (i) intact plants were taken through a drying-rewatering cycle, and (ii) detached leaves were bench-dried and subsequently xylem-perfused with AQPs inhibitors.

## Materials and Methods

### Plant material and growing conditions

All experiments were carried out with *P. trichocarpa* clone 664042 cuttings (mother tree planted in Lotbinière, Québec, Canada from a 1973 IUFRO progeny collection in Washougal, Oregon, USA). Rooted cuttings were produced and established in a greenhouse at the University of Alberta for 2 months in 3.8 L containers with sunshine mix 4 (Sun Gro Horticulture Canada Ltd.) under semi-controlled conditions (22/20°C day: night cycle, 18/6 h light: dark, watered daily, and fertilized (2g L-1 NPK15-30-15) once a week).

### Leaf hydraulic conductance measurements

Leaf hydraulic conductance was measured using the evaporative flux method (29) on six plants per treatment. A filtered (0.2 µm) 20 mM KCl+1 mM CaCl_2_ solution (subsequently referred to as ‘artificial xylem sap’, AXS) was used for these measurements. Flow rate through leaves was measured with a balance (model CP 224S, Sartorius, Göttingen, Germany), which logged data every 30 s to a computer. The air was well stirred by a fan as explained by Sack & Scoffoni [29]. Leaves were illuminated with ∼1000 µmol m^−2^ s^−1^ photosynthetically active radiation (PAR) at the leaf surface by an LED worklight (Husky, distributed by Home Depot, Atlanta, GA, USA). Leaf temperature was monitored by a thermocouple. Leaf water potential (Ψ_leaf_) was measured using a pressure chamber (PMS Instruments, Albany, OR, USA). For hydrated leaves, the *K*_leaf_ was calculated as described previously [29] using the final Ψ_leaf_ (Ψ_final_), which was determined at the end of the measurement of *E*, immediately after removing the leaf from the tubing system. The *K*_leaf_ was normalized by leaf area, which was determined with a scanner.

**Figure 1:**
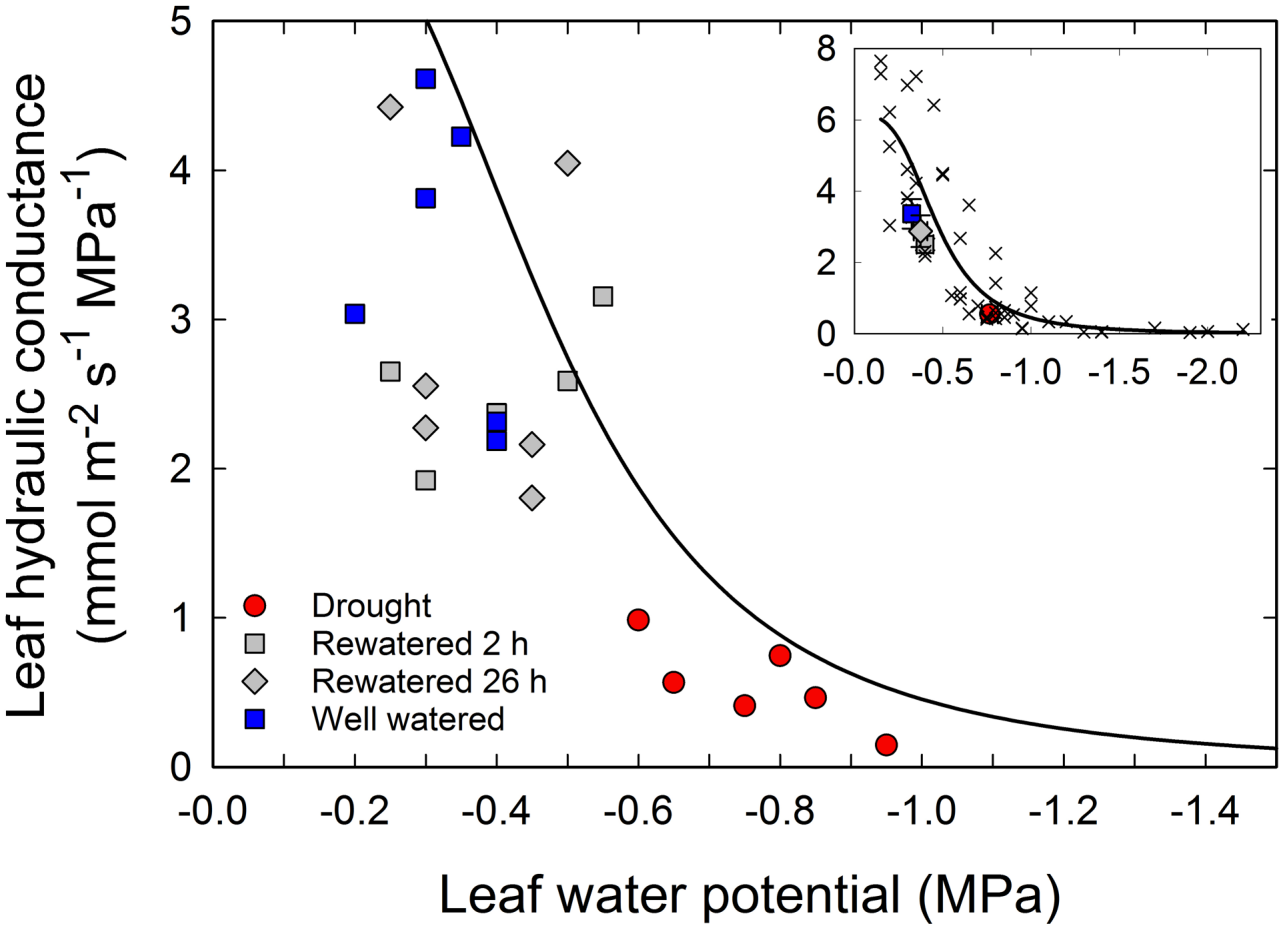
Effect of a change in water availability on leaf hydraulic conductance (*K*_leaf_) in *Populus trichocarpa* saplings. *K*_leaf_ and the associated leaf water potential (Ψ_leaf_) were measured in 6 well-watered control plants (blue squares), 6 drought-stressed plants (red circles), and drought-stressed plants 2 and 26 h after rewatering (grey squares and diamonds, respectively). Each data point represents a single measurement of *K*_leaf_. The solid line shows the previously established vulnerability curve for *K*_leaf_. A 3 parameter logistic function was used for the curve fit: *K*_leaf_ = 6.154/[1+(Ψ_leaf/_0.469)^3.337^]. An overview of the complete vulnerability curve is shown in the upper right corner of the figure. Individual measurements are shown as crosses; the mean values for each group (±SE, *n* = 6) are shown using the same symbols as explained above.

A leaf vulnerability curve was generated with plants experiencing different levels of water stress following methods of Sack & Scoffoni [29]. *K*_leaf_ was measured on leaves corresponding to leaf plastochron index (LPI) 9 [30]. The initial Ψ_leaf_ (Ψ_O_) was measured using a leaf immediately above or below the leaf that was subsequently connected to the tubing system. Once a leaf was connected to the tubing system, a stable flow rate (*E*) was usually reached in less than 20 minutes; this value of *E* was subsequently used for calculating *K*_leaf_. Leaves that did not provide stable *E* within 20 min were discarded. When dehydrated leaves are measured with the EFM, their Ψ_leaf_ may change because the petiole is connected to water at atmospheric pressure [3]. To test for this, Ψ_final_ and Ψ_O_ were compared. In most cases, Ψ_O_ and Ψ_final_ were similar and the more negative of these two values was used to calculate *K*_leaf_. When Ψ_O_ and Ψ_final_ differed by more than 0.2 MPa, the leaf was discarded and *K*_leaf_ was not calculated. The leaf vulnerability curve (shown in Fig. 1) was fitted with a 3 parameter logistic function: *K*_leaf_ = a/[1+(Ψ_leaf_/x_0_)^b^]. The curve fit was done using SigmaPlot v. 13 (Systat Software, San Jose, CA, USA). The curve fit was used to calculate the maximum leaf hydraulic conductance as well as the leaf water potentials at 50% and 80% loss of hydraulic conductance.

### Recovery of leaf hydraulic conductance after dehydration

To study the recovery of *K*_leaf_ in intact plants, plants were randomly assigned to different watering regimes in the greenhouse. One group of plants was kept well watered (control). Another group of plants was subjected to a drought treatment. Water was withheld for several days until plants reached a Ψ_leaf_ of −0.77±0.05 MPa (mean ± SE, n = 6). This Ψ_leaf_ was associated with a substantial reduction in *K*_leaf_. A subset of drought-stressed plants was then rewatered, and Ψ_leaf_ and *K*_leaf_ were re-measured 2 h and 26 h after rewatering.

To assess the effect of AQP inhibitors and abscisic acid (ABA) on the recovery of *K*_leaf_, excised leaves were bench-dried for 1 h (Ψ_leaf_ reached −1.00±0.09 MPa (mean ± SE, n = 6)) and then perfused for 2 h with AXS, AXS+0.2 mM HgCl_2_, AXS+50 mM H_2_O_2_ or AXS+50 µM ABA. Solutions were introduced into the transpiring leaf by immersing the petiole inside 50 mL containers. Leaves were placed near a fan; light was provided at a light level of ∼1,000 µmol m^−2^ s^−1^ PAR. Mercury chloride and H_2_O_2_ have been widely used as AQP inhibitors; ABA may also reduce AQP activity in leaves (reviewed in 28; 12). Control leaves were always kept hydrated and were perfused with pure AXS for 2 h. Immediately after perfusion with these solutions, *K*_leaf_ was determined as described above. All measurements were conducted at the same time of day (10∶00–11∶30 h).

After perfusion with the different solutions, stomatal pore aperture of leaves was measured as described in Laur & Hacke [31]. Images were recorded in six randomly selected fields of view of each leaf. Fields of view were located near the point of maximum leaf width on the abaxial leaf surface.

### Dye uptake experiments

The extent of dye uptake in excised leaves was used as an additional method to assess xylem refilling in leaf veins during the rehydration phase. We also used the dye uptake experiments in an attempt to study how embolism reversal in leaf veins is impacted by mercury. Excised leaves were bench-dried for 1 h and rehydrated for 2 h by immersion of the petioles in filtered safranin solutions. Transpiration during dye uptake was promoted by placing leaves near a fan at a light level of ∼1,000 µmol m^−2^ s^−1^ PAR (i.e., conditions similar to the protocol used to measure *K*_leaf_). Dye (0.1% (w/v) safranin) was dissolved in pure AXS or AXS+0.2 mM HgCl_2_. Control leaves were excised from well-watered plants and then perfused for 2 h with 0.1% safranin-containing AXS without prior dehydration treatment. Images were recorded in six randomly selected fields of view of each leaf. Fields of view were located near the point of maximum leaf width on the abaxial leaf surface.

### Gene transcript measurements by quantitative real-time PCR

Fully expanded leaves corresponding to LPI 7–10 were collected, immediately frozen in liquid nitrogen and stored at −80°C until analyzed. Samples were always collected between 10∶00 h and 11∶30 h to minimize any diurnal effect on AQP expression. Total RNA was extracted from 3 plants per treatment following the CTAB method of Pavy et al. [32]. RNA quality was assessed on an agarose gel and quantified with a spectrophotometer (Nanodrop ND-1000, Thermo Scientific, Wilmington, DE, USA). RNA was treated as previously described [19]. cDNA quality was checked by PCR with intron-spanning actin primers. Putative leaf-expressed AQP genes were selected [33]–[35], specific primers (Table S1) were designed according to Rutledge & Stewart [36] using the QuantPrime online tool [37]. PCR efficiency was 100±7% for all primer pairs and specificity was checked using melting curves. Real-time qPCR was performed on a 7900 HT Fast Real-Time PCR system (Applied Biosystems, Foster City, CA, USA) as described previously [31]. Relative gene expression was measured according to Livak & Schmittgen [38] using the 2ΔΔC(t) method. The expression values were normalized to the geometric mean of four housekeeping genes (actin (POPTR_0001s31700), cyclophilin (POPTR_0005s26170), TIP4-like (POPTR_0009s09620.1) and ubiquitin (POPTR_0005s09940)). Relative gene expression was determined as the fold change of an AQP isoform at a given condition relative to its expression under control conditions. Real-time PCR was carried out using three biological replicates each with three technical replicates.

### Immunolocalization

Samples were fixed in formaldehyde-acetic acid and embedded in paraffin as described previously [39]. Transverse sections, 10 µm thick, were prepared with a microtome. Immunoreactions were performed following the protocol of Gong et al. [40]. Primary antibodies directed against the 42 N-terminal amino acids of AtPIP1;3 [41] and the conserved 10 amino acids of the C-terminal of PIP2s [19] were used. In addition, we applied a commercially available anti-TIP2 antibody (Sakurai et al. [42]); Agrisera AB, Sweden; alignment shown in Figure S1). AlexaFluo 488-conjugated goat anti-chicken, anti-mouse and anti-rabbit secondary antibodies (Life Technologies Inc., Burlington, ON, Canada) were applied respectively for 2 h at 37°C. Slides were mounted with Permount. Images were taken with a Zeiss LSM 700 confocal microscope (Carl Zeiss, Oberkochen, Germany).

### Statistical analysis

All statistical analyses were carried out using SigmaPlot software. Differences due to the effect of physiological treatments were analyzed after testing for normality and equal variance by using a one-way ANOVA followed by a Tukey’s test. A one-way ANOVA followed by Bonferroni’s post test was used for the gene expression analysis. Differences were considered significant at *P*≤0.05.

## Results and Discussion

### Leaf hydraulic conductance is highly sensitive to drought

To assess how *K*_leaf_ declines as a function of Ψ_leaf_, we first constructed a vulnerability curve. Water was withheld from plants in the greenhouse until plants reached different levels of water stress. Leaves were highly vulnerable with 50% and 80% loss of hydraulic conductance occurring at Ψ_leaf_ = −0.47 MPa and −0.71 MPa, respectively (Figure 1, insert). According to the curve fit in Figure 1 (insert), the maximum *K*_leaf_ measured during this experiment was 6.15 mmol m^−2^ s^−1^ MPa^−1^. The drought-induced loss in *K*_leaf_ shown in Figure 1 may have been due to xylem cavitation, reduced water permeability of cell membranes and/or other factors [6], [7]. The water potentials at 50% and 80% loss of hydraulic conductance (*P*_50_ and *P*_80_, respectively) are well within the range of water potentials that trees experience under natural conditions [43], [44]. It therefore appears that *K*_leaf_ is subject to substantial diurnal changes under natural conditions, similar to what has been observed in rice and other species [3], [15], [45], [46]. Our data also indicates that leaf hydraulic conductance is more sensitive to decreasing water potentials than the hydraulic conductance of stems [43]. However, since we only worked with young greenhouse-grown plants, it remains to be seen whether leaves of field-grown trees are similar in their response to water stress.

### Leaves of intact plants quickly recover from drought

We next tested whether *K*_leaf_ would recover after a drought treatment when plants were left intact during the dehydration-rehydration episode. In this experiment, leaves of well-watered control plants had a Ψ_leaf_ of −0.33±0.03 MPa (±SE, n = 6), which was associated with a *K*_leaf_ of 3.37±0.41 mmol m^−2^ s^−1^ MPa^−1^ (±SE, n = 6) (Figure 1, blue squares). The drought treatment resulted in a drop of Ψ_leaf_ to −0.77±0.05 MPa (± SE, n = 6) and a six-fold drop of *K*_leaf_ to 0.55±0.12 mmol m^−2^ s^−1^ MPa^−1^ (±SE, n = 6) (Figure 1, red circles). These values were in good agreement with the previously established vulnerability curve (Figure 1, insert). Only 2 h after rewatering (Figure 1, grey squares), both Ψ_leaf_ and *K*_leaf_ reached values that were not statistically different from well-watered control plants (*t* test, *P* = 0.083 for *K*_leaf_), indicating that leaves completely recovered their hydraulic function.

### *AQP* expression in leaves collected from intact plants

To study the role of water channels in the recovery of *K*_leaf_, AQP expression was measured in leaves at different stages during the dehydration-rehydration experiment. Three *PIP1*, three *PIP2,* and six *TIP* candidate genes were selected for analysis. Among them, *PtPIP1;1*, *PtPIP1;2*, *PtPIP1;3*; *PtPIP2;4* and *PtTIP2;1* exhibited the highest total number of mRNA molecules in leaves of control plants (Table 1).

**Table 1:** Transcript abundance of 12 aquaporin genes expressed in leaves of well-watered control plants.

Aquaporin name	Expression (copies µg^−1^ of total RNA)
PtPIP1;1	112,960±9,067
PtPIP1;2	272,111±32,575
PtPIP1;3	229,960±44,252
PtPIP2;3	85,667±15,402
PtPIP2;4	273,655±33,728
PtPIP2;5	11,536±1,738
PtTIP1;3	23,105±2,540
PtTIP1;5	11,840±1,675
PtTIP1;6	2,330±121
PtTIP2;1	153,689±19,669
PtTIP2;2	24,863±3,451
PtTIP4;1	517±9

Values are the means ± SE from three biological samples which were tested in triplicate.

**Figure 2:**
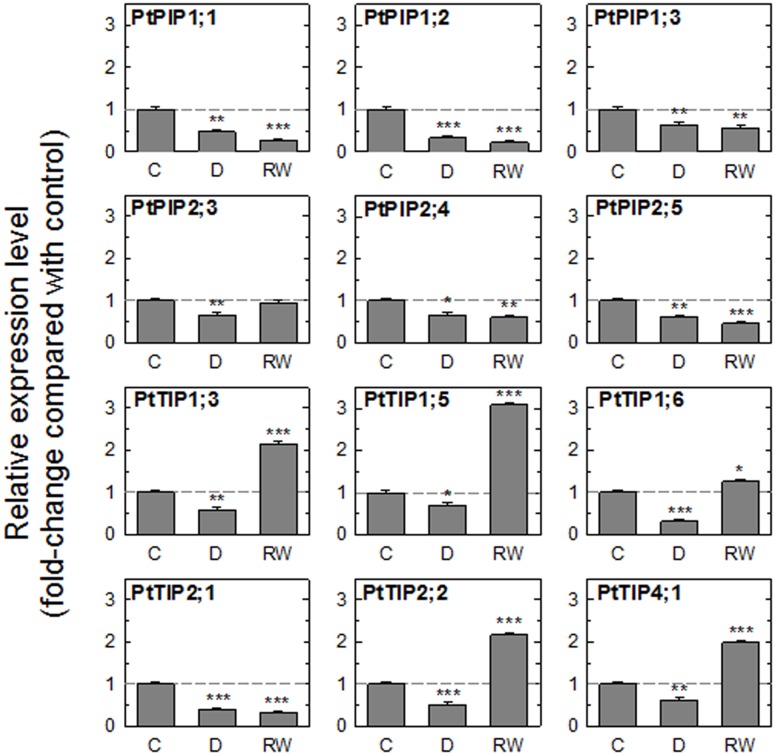
Relative expression of aquaporin genes in leaves of plants exposed to a drying-rewatering cycle. Gene expression was measured in leaves of well-watered control plants (C), drought-stressed plants (D), and 3 h after drought-stressed plants were rewatered (RW). The geometric mean of the expression levels of four reference genes (*ACT2*, *CYC063*, *TIP41-like*, *UBQ7*) was used to normalize the results. Asterisks denote significant differences in expression level compared to control levels (one-way ANOVA, followed by Bonferroni’s post test, *P≤0.05; **P≤0.01***P≤0.001). Data are means ± SE of three biological replicates.

The drought treatment resulted in a significant reduction in the expression of all tested genes (Fig. 2). In leaves collected 2 h after rewatering, there were two patterns of expression between the 12 isoforms. One group of genes (among them all *PIP1*s) remained down-regulated while the expression of a second group of genes matched or exceeded the transcript levels measured in control leaves. With the exception of *PtTIP2;1*, all tested *TIP*s were significantly up-regulated after 2 h. Among the *PIP*s, only the expression level of *PtPIP2;3* increased to match the control level.

### Recovery of *K*_leaf_ in detached leaves is impaired by inhibitors

**Figure 3:**
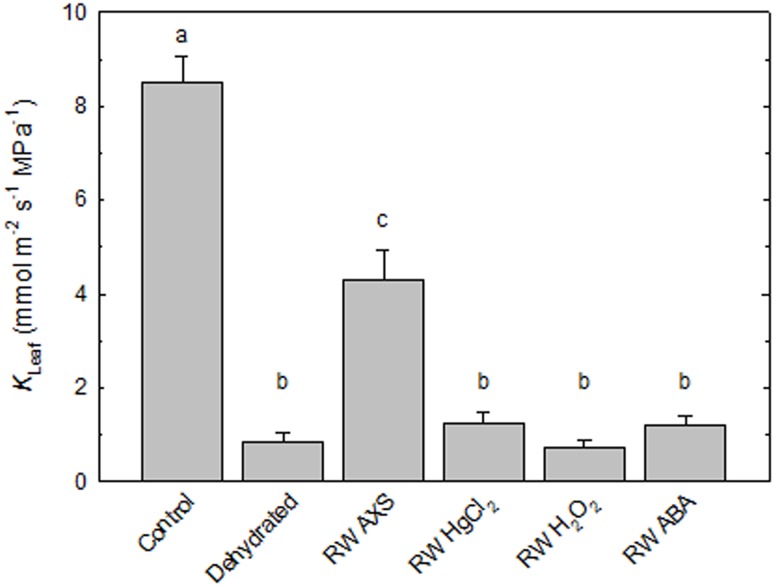
Response of leaf hydraulic conductance (A) and stomatal aperture (B) to different perfusion solutions. Control conditions refer to the *K*_leaf_ that was measured after leaves were xylem perfused with filtered (0.2 µm) 20 mM KCl+1 mM CaCl_2_ solution (subsequently referred to as ‘artificial xylem sap’, AXS) for 2 h. *K*_leaf_ was also measured on leaves that were bench-dried for 1 h (Dehydrated) and on leaves that were bench-dried for 1 h and subsequently perfused for 2 h with AXS (RW AXS), AXS+0.2 mM HgCl_2_ (RW HgCl_2_), AXS+50 mM H_2_O_2_ (RW H_2_O_2_) or AXS+50 µM ABA (RW ABA). Values are means ± SE (n = 6). Different letters denote statistically significant differences by one-way ANOVA with Tukey’s test.

Another set of experiments was conducted on leaves that were excised from the plant prior to the dehydration-rehydration treatment. Working with detached leaves allowed us to study the effect of AQP inhibitors and ABA on the recovery of *K*_leaf_. Control leaves exhibited a *K*_leaf_ of 8.49±0.57 mmol m^−2^ s^−1^ MPa^−1^ (±SE, n = 6), which is higher than the values shown in Figure 1. One difference between the data shown in Figures 1 and 3 is that all data in Figure 1 was derived from leaves that were excised (petioles were cut under water) from transpiring plants immediately before *K*_leaf_ was measured while the control leaves in Figure 3 were perfused with AXS for 2 h prior to measuring *K*_leaf_. Hence, the absolute *K*_leaf_ values shown in Figures 1 and 3 are not readily comparable.

Bench-drying of leaves caused a ∼10-fold decline in *K*_leaf_ relative to fully hydrated control leaves (Figure 3A). Dehydrated leaves that were subsequently xylem-perfused for 2 h with AXS exhibited a significant recovery to 50% of the hydraulic conductance measured in control leaves. The fact that recovery remained incomplete in detached leaves is consistent with an involvement of phloem transport in embolism repair [47], [48]. Application of commonly used inhibitors allowed us to assess the impact of AQPs on *K*_leaf_ during leaf rehydration. Leaves fed with HgCl_2_ and H_2_O_2_ did not exhibit any recovery of hydraulic conductance, indicating that AQPs were involved in the recovery of *K*_leaf_ after dehydration. A role of AQPs in embolism repair has also been proposed for other species and plant organs [18], [19], [49]–[51].

We next asked whether differences in *K*_leaf_ were associated with different degrees of stomatal closure. Stomatal apertures in fully hydrated control leaves were 9.5±0.1 µm (±SE, n = 6), similar to the value of ∼10 µm previously reported for *P. trichocarpa* leaves [52]. Schulte and Hinckley [52] found that stomatal aperture in this species was not affected by a wide range of epidermal water potentials. Our data supports these findings as we also did not observe complete stomatal closure in any of our experimental treatments (Figure 3B). Even in dehydrated leaves and in leaves that were perfused with AQP inhibitors and ABA, stomatal aperture remained at ∼6 µm. This value is similar to the maximum apertures found in *P. deltoides* and in *P. trichocarpa* x *deltoides* hybrids [31], [52]. We conclude that the magnitude of the decline in *K*_leaf_ in dehydrated leaves and in leaves that were perfused with AQP inhibitors is greater than that of changes in stomatal aperture. Guyot et al. [53] also found a discrepancy between patterns of stomatal conductance and *K*_leaf_, and they discuss possible reasons for a mechanistic independence of stomatal and leaf hydraulic conductance.

**Figure 4:**
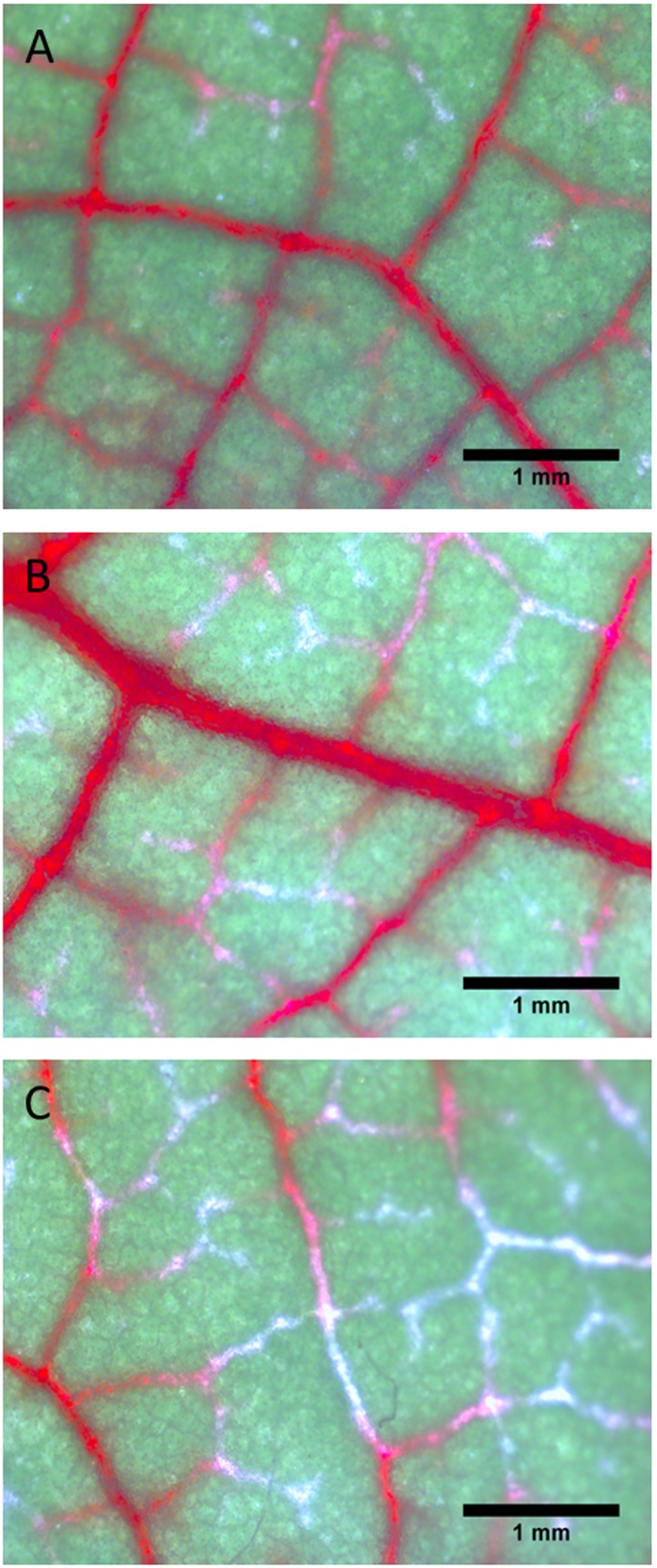
Typical images of transpiring *P. trichocarpa* leaves that were allowed to take up safranin solution. (A) A control leaf was excised from a well-watered plant, and the petiole was immersed for 2 h in safranin solution. Transpiration during dye uptake was promoted by placing the leaf near a fan at ∼1,000 µmol m^−2^ s^−1^ photosynthetic active radiation. Most leaf veins were stained indicating minimal xylem embolism. (B) Dye uptake in a bench-dried leaf that was subsequently perfused with safranin solution for 2 h. Minor veins exhibited incomplete staining indicating the presence of embolized xylem conduits in minor veins. (C) Dye uptake of a bench-dried leaf subsequently perfused with safranin + HgCl_2_ solution for 2 h. Mercury is an aquaporin inhibitor. Staining remained even more incomplete than in (B).

We used the dye uptake experiments in an attempt to study how embolism reversal in leaf veins is impacted by mercury. Nearly all veins of well-watered control leaves were stained and functional (Figure 4A). In leaves that were bench-dried and subsequently supplied with ASX + safranin for 2 h, many minor veins exhibited incomplete staining (Figure 4B). Staining was even less complete in leaves that were bench-dried and subsequently perfused with ASX + safranin + HgCl_2_ (Figure 4C).

These findings suggest that embolism formation in minor veins had a substantial impact on the dynamics of *K*_leaf_. Studying water transport in rice leaves, Stiller et al. [8] reported that the leaf xylem experienced high embolism levels, even in watered controls. Nardini et al. [54] found that minor veins of *Cercis siliquastrum* leaves underwent extensive embolism at leaf water potentials <−1.5 MPa, indicating that leaf vein embolism was closely related to *K*_leaf_ changes. Recently, Johnson et al. [55] suggested that reductions in *K*_leaf_ are directly related to vein embolism. On the other hand, a recent study found that hydraulic decline during mild dehydration was associated with leaf shrinkage [10]. The changes in *K*_leaf_ we observed in this present study were likely caused by xylem and extra-xylem components; it is difficult to determine the relative importance of either component. In addition, both xylem refilling in leaf veins and the permeability of extra-xylem tissues may be impacted by AQP function.

### *AQP* expression in detached leaves

**Figure 5:**
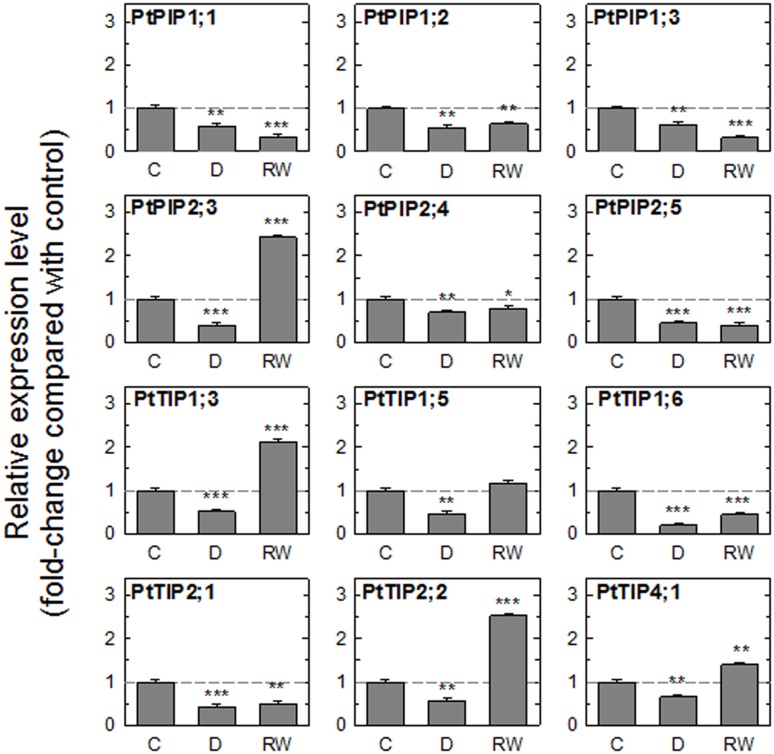
Relative expression of aquaporin genes in detached leaves during a dehydration-rehydration experiment. Data are from control leaves (C) after they were perfused with artificial xylem sap (AXS) for 2 h, leaves that were dehydrated on the bench top for 1 h (D), and leaves that were dehydrated on the bench top for 1 h and then perfused for 2 h with AXS (RW). The geometric mean of the expression levels of four reference genes (*ACT2*, *CYC063*, *TIP41-like*, *UBQ7*) was used to normalize the results. Asterisks denote significant differences in expression level compared to control levels (one-way ANOVA, followed by Bonferroni’s post test, *P≤0.05; **P≤0.01***P≤0.001). Data are means ± SE of three biological replicates.

Aquaporin expression was measured in detached leaves undergoing a dehydration-rehydration cycle (Figure 5). Control leaves were perfused with AXS for 2 h before leaf tissue was sampled for the gene expression analysis. As previously seen in intact plants (Figure 2), water stress caused down-regulation of all tested *AQP*s (Figure 5). This agrees with several previous studies [19], [56], [57].

**Figure 6:**
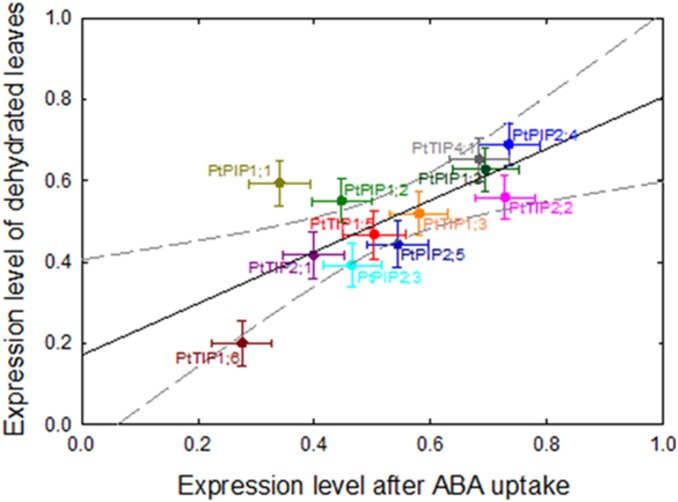
Relative expression of 12 aquaporin genes in response to dehydration (y-axis) and dehydration + perfusion with abscisic acid (x-axis). Detached leaves were either dehydrated on the bench top for 1 h or dehydrated for 1 h and subsequently perfused for 1 h with 50 µM abscisic solution (ABA). Data from fully hydrated detached leaves (perfused for 3 h with 20 mM KCl+1 mM CaCl_2_ solution) were used as the control group, and their expression refers to a value of 1. Pearson’s r = 0.725; *P*≤0.01. Data are means ± SE of three biological replicates.

Notably, very similar degrees of down-regulation were found in bench-dried leaves and in dried leaves that were subsequently xylem-perfused with AXS + ABA (Figure 6, r = 0.725, *P*<0.01). Genes that were strongly down-regulated by dehydration, such as *PtTIP1;6* also exhibited strong down-regulation after perfusion with ABA solution while the expression of other genes, such as *PtPIP2;4*, changed less in response to either of these factors (Figure 6). Excluding *PtPIP1;1* from the analysis shown in Figure 6 further increased the strength of the linear relationship (r = 0.89, *P*<0.001).

The lack of recovery in ABA-perfused leaves and down-regulation of *AQP*s in leaves supplied with AXS + ABA is consistent with the model of Shatil-Cohen et al. [12]. Working with *Arabidopsis*, these authors also used a ‘detached leaf’ approach to feed ABA to the xylem via the petiole. Feeding the leaf with ABA decreased *K*_leaf_ by nearly 50%. In contrast, smearing ABA on the leaf surface, while reducing transpiration, had no effect on *K*_leaf_. Shatil-Cohen et al. [12] proposed that the membrane water permeability of bundle sheath cells is controlled by AQPs, and that the bundle sheath would act like a control center regulating *K*_leaf_ in response to signals from the xylem. As the concentration of ABA increases in the xylem, AQP activity in the bundle sheath would be down-regulated, reducing water flow into the leaf mesophyll. Bundle sheath cells, and perhaps xylem parenchyma cells, seem to have a specific responsiveness to ABA, which likely explains the negative effects of this hormone on *K*_leaf_ (for a recent review see 7). While our data is consistent with these observations, it is not clear yet which cells may perform the role of a ‘control center’ in *P. trichocarpa* leaves (Figure S2). While we previously observed prominent PIP1 and PIP2 labeling of the endodermis-like bundle sheath in *Picea glauca* needles [19], no such pattern was found in this present study.

**Figure 7:**
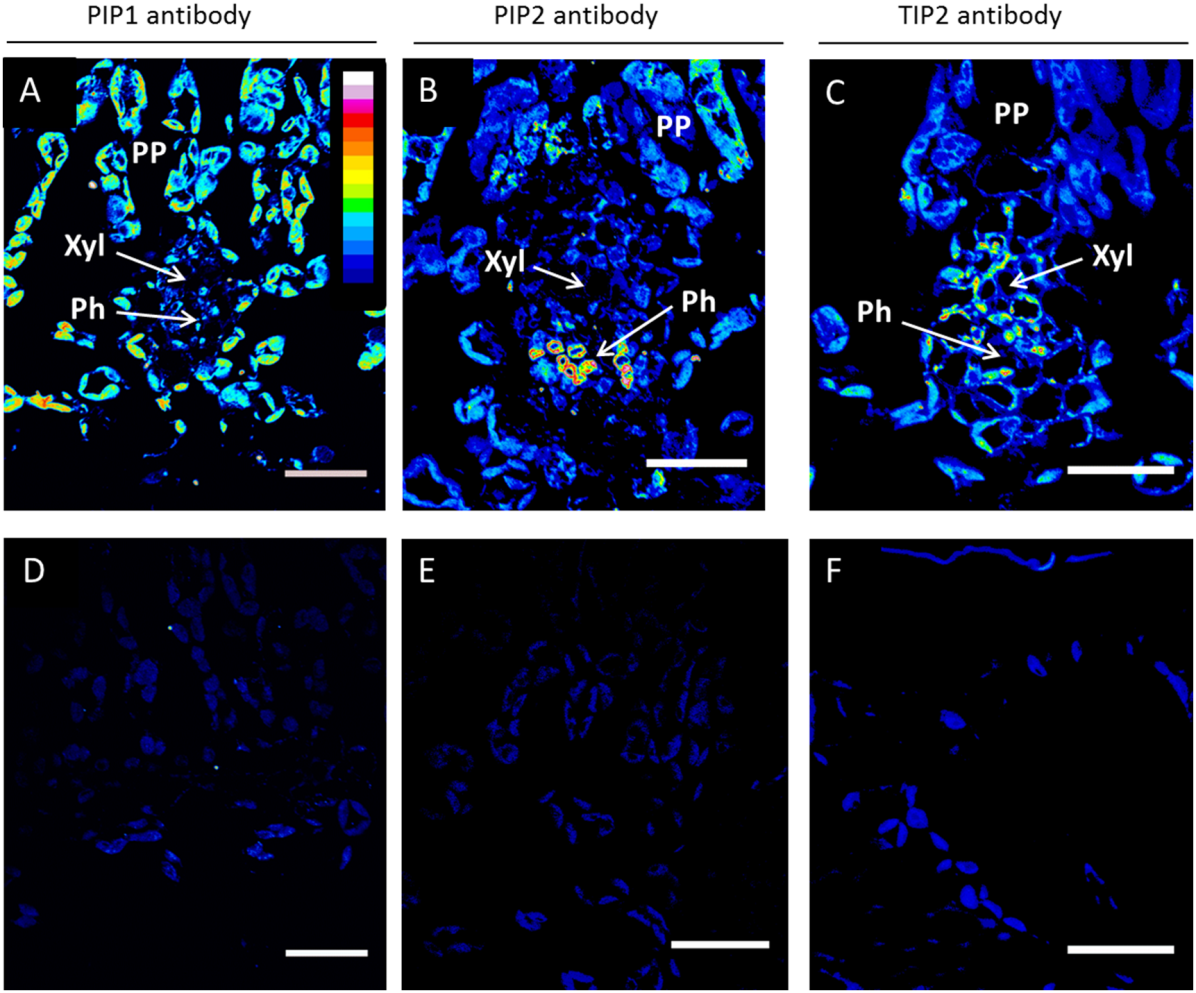
Immunolocalization of AQP proteins in leaves of *P. trichocarpa* saplings. Confocal laser scanning micrographs showing the localization of PIP1, PIP2, TIP2 proteins in minor veins of leaf transverse sections (A, B, C respectively). Controls with no primary antibody indicate minimal background fluorescence (D, E, F respectively). Images were taken at an identical setting and were color-coded with an intensity look-up-table (LUT; displayed in A), in which black was used to encode background, and blue, green, yellow, red and white to encode increasing signal intensities. Ph, phloem; PP, palisade parenchyma; Xyl, xylem. Scale bars = 20 µm.

In rehydrated leaves, four genes showed increased expression levels relative to control leaves (Figure 5). Three of these AQPs (*PtTIP1;3*, *PtTIP2;2*, and *PtTIP4;1*) were TIPs and were also found to be up-regulated when intact plants were rewatered after a drought (compare Figures 2 and 5). While TIPs have rarely been studied in the context of water flow through tissues and embolism repair, a recent study on grapevine plants found a striking positive correlation between *K*_leaf_ and the transcript abundance of *VvTIP2;1* [58]. Our immunolocalization experiments indicate that TIP2 protein was present in xylem parenchyma cells (Figure 7). This agrees with the expression pattern of *ZmTIP1* in leaves and stems of maize. *In situ* localization revealed that this tonoplast AQP was highly expressed in parenchyma cells surrounding xylem vessels, in phloem companion cells, and between the phloem and the xylem strands [59]. Barrieu et al. [59] hypothesized that the high expression of the ZmTIP1 tonoplast AQP in xylem parenchyma cells would allow these cells to control water movement in and out of the xylem vessels. Daniels et al. [60] found that AtTIP2 expression in mature leaves was generally restricted to vascular tissues. In stem xylem of hybrid poplar, a TIP2 AQP was highly expressed in contact cells, suggesting a role in increasing water exchange between vessels and xylem rays [61].

In this present study, we also determined the cell- and tissue-level localization of PIP1 and PIP2 proteins (Figure 7). All sections were taken from leaves of well-watered plants. Strong PIP1 signals were present in the palisade parenchyma (Figure 7A). PIP1 antibody was also detected in vein cells, including phloem and xylem parenchyma. This labeling pattern is consistent with a dual role of PIP1s in influencing permeability to water and CO_2_ [62]. PIP2 was mostly localized in the phloem, which agrees with previous studies [19], [27], [61], [63], [64]. Weaker PIP2 labelling was evident in palisade parenchyma cells (Figure 7B).

## Conclusions

We studied how AQPs may be involved in the recovery of water stress-induced declines in *K*_leaf_. We examined how *K*_leaf_ responds to known AQP inhibitors and xylem-fed ABA. We also examined the expression of 12 highly expressed AQP genes during dehydration-rehydration experiments. Hydraulic measurements and gene expression assays were complemented by dye uptake and immunolocalization experiments. This has revealed that, while *P. trichocarpa* leaves are highly sensitive to dehydration, leaf hydraulic conductance can quickly recover when water becomes available again. Recovery of *K*_leaf_ was absent when excised leaves were xylem-perfused with AQP inhibitors, suggesting that the recovery of leaf hydraulic function is associated with AQP activity. Among the AQPs tested, several *TIP*s showed large increases in expression in rehydrated leaves, suggesting that TIPs play an important role in reversing drought-induced reductions in *K*_leaf_.

## Supporting Information

Figure S1 (a) Amino acid multiple sequence alignment of the N-terminal region of the *Arabidopsis thaliana* AtPIP1;3 and the *Populus trichocarpa* PtPIP1s; (b) of the conserved the C-terminal region of PIP2s, and (c) TIP2s. Consensus amino acids are underlined in black.
(DOCX)

Figure S2 **Transverse section of a** ***Populus trichocarpa*** **leaf showing minor veins with (left) and without (right) bundle sheath cell extensions.** Scale bar = 20 µm.
(DOCX)

Table S1 **Primer sequences used for the gene expression study.**
(DOCX)
