# Supplementary material for: The Role of Water Channel Proteins in Facilitating Recovery of Leaf Hydraulic Conductance from Water Stress in Populus trichocarpa
Source: PLoS One. 2014 Nov 18;9(11):e111751. doi: 10.1371/journal.pone.0111751 (PMC4236056; doi:10.1371/journal.pone.0111751)
Supplement: Figure S1 — (a) Amino acid multiple sequence alignment of the N-terminal region of the Arabidopsis thaliana AtPIP1;3 and the Populus trichocarpa PtPIP1s; (b) of the conserved the C-terminal region of PIP2s, and (c) TIP2s. Consensus amino acids are underlined in black. (DOCX) [file pone.0111751.s001.docx]

**Figure S1**

**a** **antiPIP1** ME-GKEEDVRVGANKFPERQPIGTSAQS-D-KDYKEPPPAPFFEP

PtPIP1;1 ME-GKEEDVRLGANKFNERQPLGTAAQSQDDKDYKEPPPAPLFEP

PtPIP1;2 ME-GKEEDVRLGANKFNERQPIGTAAQSLDDKDYKEPPPAPLFEP

PtPIP1;3 ME-GKEEDVKLGANKFSERQPIGTSAQ-TD-KDYKEAPPAPLFEP

PtPIP1;4 MEEG-EEDVKVGANRYGEGQPIGTAAQTQHGKDYTEPPPAPLYQP

PtPIP1;5 ME-GREEDVRVGANKYGERQPIGTAAQAQDVKDYTDPPPAPLFEP

**b antiPIP2** K---ALGSFRS---NP

PtPIP2;1 K---ALGSFRSHPTN

PtPIP2;2 K---ALGSFRS---NP

PtPIP2;3 K---ALGSFRSAQRF

PtPIP2;4 K---ALGSFRS---NP

PtPIP2;5 K---ALGSFRSSS-N

PtPIP2;7 K---SLGSFRSS--PN

PtPIP2;8 K---ALGSFRS---NA

PtPIP2;9 K---ALASFRS---NP

PtPIP2;10 KSFRALGSFGSQP--P

**c antiTIP2** CGDHAP--VASS-EF

PtTIP2;1 CTDHTP----LSGDF

PtTIP2;2 CTDHSP----SSYEF

PtTIP2;3 IGSYAPAPV--S-ED

PtTIP2;4 IGSYTAAPV--S-ED
