# Supplementary material for: The Role of Water Channel Proteins in Facilitating Recovery of Leaf Hydraulic Conductance from Water Stress in Populus trichocarpa
Source: PLoS One. 2014 Nov 18;9(11):e111751. doi: 10.1371/journal.pone.0111751 (PMC4236056; doi:10.1371/journal.pone.0111751)
Supplement: Figure S2 — Transverse section of a Populus trichocarpa leaf showing minor veins with (left) and without (right) bundle sheath cell extensions. Scale bar = 20 µm. (DOCX) [file pone.0111751.s002.docx]

**Figure S2**


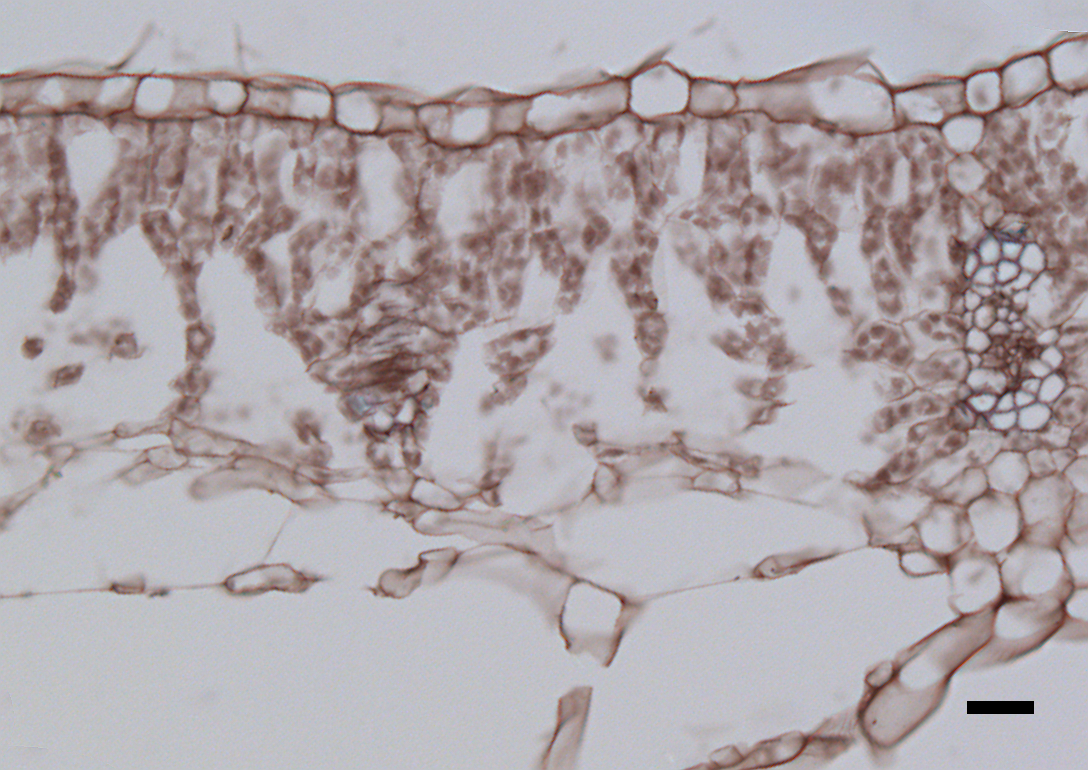


Bundle sheath cell extension

Xylem

Phloem

Upper epidermis

Lower epidermis

Spongy parenchyma

Palisade parenchyma

Bundle sheath cell extension


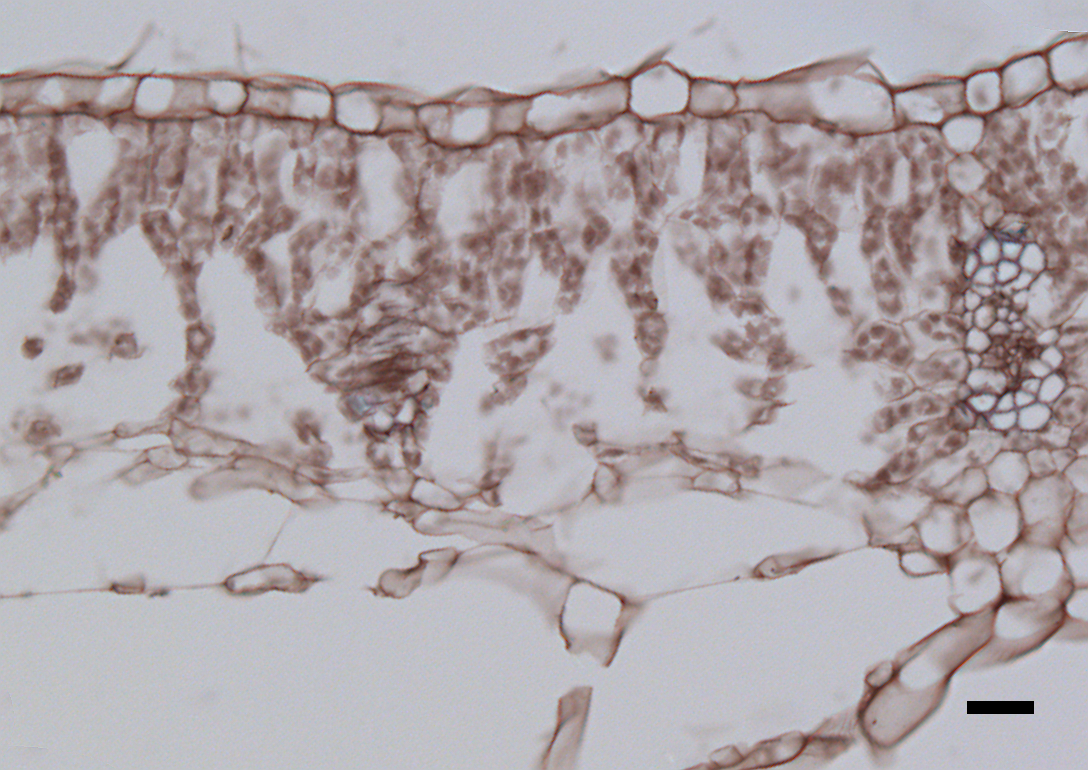


Bundle sheath cell extension

Xylem

Phloem

Upper epidermis

Lower epidermis

Spongy parenchyma

Palisade parenchyma

Bundle sheath cell extension
