# Supplementary material for: The Role of Water Channel Proteins in Facilitating Recovery of Leaf Hydraulic Conductance from Water Stress in Populus trichocarpa
Source: PLoS One. 2014 Nov 18;9(11):e111751. doi: 10.1371/journal.pone.0111751 (PMC4236056; doi:10.1371/journal.pone.0111751)
Supplement: Table S1 — Primer sequences used for the gene expression study. (DOCX) [file pone.0111751.s003.docx]

**Table S1**

|  |  | | Amplicons | | | |
| --- | --- | --- | --- | --- | --- | --- |
|  |  | | Forward Primer (5´→3´) | | Reverse Primer (5´→3´) | Length (bp) |
|  |  | |  |  |  |  |
|  |  | |  |  |  |  |
| PtPIP1;1 | | POPTR_0010s19930 | | TGCAGAGTTCATGGCCACCTTC | TCGTGTCCTTAAACACGCCCATC | 74 |
|  |  |  |  |  |  |  |
| PtPIP1;2 | | POPTR_0008s06580 | | TGGCCTTGGTGCTGAGATTGTC | GCACTACGCTTGGCATCAGTTG | 78 |
|  |  |  |  |  |  |  |
| PtPIP1;3 | | POPTR_0003s12870 | | AACTGGCATTAACCCGGCAAGG | AATGGGCCAACCCAGAAGATCCAG | 96 |
|  |  |  |  |  |  |  |
| PtPIP2;3 | | POPTR_0010s22950 | | AGTCTGGGAGCCGCTGTTATCTAC | GGGTCCAACCCAGAAGATCCAATG | 72 |
|  |  |  |  |  |  |  |
| PtPIP2;4 | | POPTR_0008s03950 | | GTCATTCAGGAGCAACCCGAATGTC | CCATCATGCACGCACAAGCACTC | 81 |
|  |  |  |  |  |  |  |
| PtPIP2;5 | | POPTR_0006s12980 | | TGTGTTGGCACCACTTCCCATC | GTCATCCCATGCCTTGTCTTCGT | 139 |
|  |  |  |  |  |  |  |
| PtTIP1;3 | | POPTR_0010s21700.1 | | TTCAGGATCTGGCATGGCTTTCAAC | CCAGAAGGAGTAGTCGAAGCATTGTCG | 60 |
| PtTIP1;5 | | POPTR_0016s10780.1 | | TCCACTGTCGCTTGCTTGCTTC | ACAGAGCGAAAGCAGAGGTTTCCAAG | 67 |
| PtTIP1;6 | | POPTR_0006s12350.1 | | TCCACTGTCGCTTGCTTGCTTCTC | ACAGAGCGAAAGCAGAGGTTTCCAG | 67 |
| PtTIP2;1 | | POPTR_0001s18730.1 | | GCCATGGCTTACAATAAGCTGACAGGTG | GGCACCTACAGAAACTGCAACGAAG | 111 |
| PtTIP2;2 | | POPTR_0003s04930.1 | | TGGCTTACAATAAGCTGACAGGTGATGC | ACCAACAGCAACTGCAACAAAGAGC | 104 |
| PtTIP4;1 | | POPTR_0006s25620.1 | | TCAAGTATCTCACCGGAGGATTGGC | CCTTGAAGGTAGTCCATCCCACTTGC | 70 |
| ACT | | POPTR_0001s31700 | | TGGAGGATCTATCCTTGCTTCCCTCAG | TACTCACCCTTGGAAATCCACATCTGC | 63 |
| CYCL | | (POPTR_0005s26170 | | ACCAGGTAAGCAAGCGGTTTGGTC | TCGACCGATTTCCATGGAGTGCAAG | 72 |
| TIP4 | | POPTR_0009s09620.1 | | AGAGTCATGCCAAGTTGCTGGTTTC | TCGACCGATTTCCATGGAGTGCAAG | 60 |
| UBQ | | POPTR_0005s09940 | | TCCACCTGTGCAACAAAGGC | CACTCCATCAACTCTAAGCCAGAATCGC | 66 |
